# Protective Efficacy and Mechanism of Passive Immunization with Polyclonal Antibodies in a Sepsis Model of *Staphylococcus aureus* Infection

**DOI:** 10.1038/srep15553

**Published:** 2015-10-22

**Authors:** Jinyong Zhang, Feng Yang, Xiaoli Zhang, Haiming Jing, Chunyan Ren, Changzhi Cai, Yandong Dong, Yudong Zhang, Quanming Zou, Hao Zeng

**Affiliations:** 1 National Engineering Research Center of Immunological Products, Department of Microbiology and Biochemical Pharmacy, College of Pharmacy, Third Military Medical University, Chongqing, 400038, PR China; 2 College of Bioengineering, Chongqing University, Chongqing, 400044, PR China; 3 Department of Clinical Hematology, Southwest Hospital, Third Military Medical University, Chongqing, 400038, PR China

## Abstract

*Staphylococcus aureus* (*S. aureus*) is an opportunistic bacterial pathogen responsible for a diverse spectrum of human diseases, resulting in considerable yearly mortality rates. Due to its rapid acquisition of antibiotic resistance, it becomes increasingly difficult to cure *S. aureus* infections with conventional antibiotics. Immunotherapy represents a promising alternative strategy to prevent and/or treat the infection. In the present study, passive immunization with polyclonal antibodies targeting three possible *S. aureus* antigens, Hla, SEB and MntC (termed “SAvac-pcAb”) after challenge with lethal dose of *S. aureus* resulted in reduced bacterial loads, inflammatory cell infiltration and decreased pathology, and was able to provide nearly complete protection in a murine sepsis model. *In vitro* studies confirmed the direct interaction of SAvac-pcAb with *S. aureus* bacteria. Additional studies validated that SAvac-pcAb contained both opsonic and neutralizing antibodies that contributed to its protective efficacy. The former mediated opsonophagocytosis in a neutrophil-dependent manner, while the later inhibited the biological functions of Hla and SEB, two major virulence factors secreted by *S. aureus.* Critically, we demonstrated that SAvac-pcAb was cross-reactive with different clinical strains of *S. aureus.* These results confirmed the efficacy for treatment of *S. aureus* infection by passive immunization as an important therapeutic option.

*Staphylococcus aureus* (*S. aureus*) is an opportunistic bacterial pathogen that is responsible for a variety of superficial and invasive infectious diseases in human, including soft tissue infection, bacteremia, endocarditis, pneumonia, sepsis, and general wound infections^1^. Such infections are associated with considerable morbidity and mortality, both in hospitals and the greater community, thereby posing a major global health challenge^2^. In addition, the emergence of drug-resistance strains, such as methicillin-resistant *S. aureus* (MRSA) and vancomycin-resistant *S. aureus* (VRSA), make it increasingly difficult to cure the infection^3^.

Immunotherapy represents a promising strategy to prevent *S. aureus* related infectious diseases^4^,^5^. Efforts to develop an effective vaccine against *S. aureus* infection have been ongoing, with extensive studies currently underway^6^. A wide variety of proteins from *S. aureus* were identified as promising candidate antigens, including capsular polysaccharides^7^, secreted toxins^8^, and out membrane proteins^9^. In previous studies, we reported three proteins that exhibited protective immunity against *S. aureus* infection, including a genetically detoxified staphylococcal alpha-toxin mutant H35L (mHla)^10^, staphylococcal enterotoxin B mutant L45R/Y89A/Y94A (mSEB)^11^ and wild-type manganese transport protein C (MntC) (submitted). Active immunization with either of these proteins was able to induce specific antibodies and cellular immune responses, resulting in reduced bacterial loads and inflammation reaction, as well as improved survival time and rate in mice.

However, *S. aureus* usually causes an acute infection with rapid progression, and 60% of patients with invasive infections die within 7 days of culturing positive for MRSA^12^, indicating that active immunization is not the best choice for the prevention of such acute infections. Contrastingly, passive immunization is able to provide immediate and effective protection, as previous studies have demonstrated that antibody responses play a major protective role in specific immunity against MRSA^13^, and passive immunization with antigen specific antibody is able to provide partial protection against *S. aureus* infections^14^,^15^. Thus, in this study, we have systematically evaluated the protective efficacy of passive immunization with rabbit-generated polyclonal antibodies against mHla, mSEB, and MntC (termed “SAvac-pcAb”) in a murine sepsis model, and further investigated the possible mechanisms that might contribute to its protective immunity.

## Results

### Rabbit-generated pcAbs recognize recombinant proteins and sonicated MRSA252 whole cell lysates with high IgG titer

**Figure 1:**
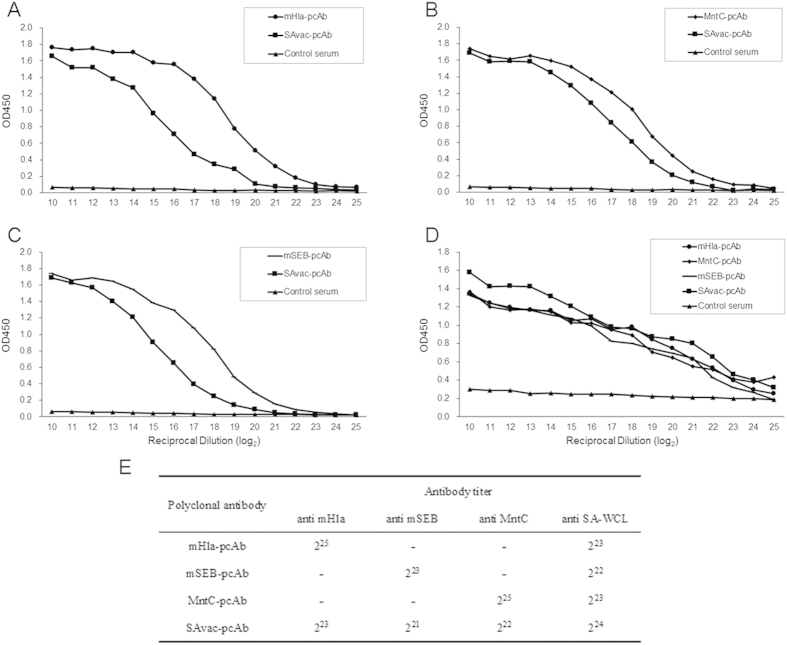
Characterization of pcAb generated from rabbits immunized with mHla, mSEB, MntC, and SAvac. ELISA was used to determine the titer of antigen-specific IgG for the indicated pcAbs. In brief, 96-well plates were pre-coated with mHla (**A**), mSEB (**B**), MntC (**C**), and sonicated MRSA252 whole cell lysates (SA-WCL) (**D**). The concentration of purified rabbit-raised antibodies was adjusted to 20 mg/ml and then added to the well by a two-fold serial dilution (2^10^ to 2^25^). The OD450 from each dilution was then determined. Data were presented as the mean from two separate experiments, each individually conducted in triplicate. (**E**) Presented as the titer of antigen-specific IgG antibody for each pcAb, which was defined as the highest dilution giving an absorbance value of more than twice that of the blank control.

In this study, pcAbs against mHla, mSEB, MntC, and SAvac (named as mHla-pcAb, mSEB-pcAb, MntC-pcAb, and SAvac-pcAb, respectively) were generated in New Zealand white rabbits and purified. These PcAbs were further characterized by the titers of specific IgG antibodies against different recombinant proteins as well as sonicated MRSA252 whole cell lysates (SA-WCL). As shown in Fig. 1, these pcAbs interacted with immunized proteins with high titer, ranging from 2^21^ to 2^25^. Both mHla and MntC exhibited a similar antibody-induction response (2^25^), which was four-fold higher when compared to mSEB (2^23^). Furthermore, SAvac-pcAb was able to react with all the three proteins, with an IgG titer that was four- to eight-fold lower when compared to pcAb against a single protein. More importantly, pcAbs that generated by recombinant proteins were also able to recognize SA-WCL, with a titer range from 2^22^ to 2^24^. SAvac-pcAb exhibited a relatively high titer, as it was raised in rabbits immunized with all three antigens. Taken together, these results suggested that the pcAbs generated in this study were able to recognize *S. aureus* with high IgG titers *in vitro*.

### Passive immunization protects mice against lethal challenge of *S. aureus*

**Figure 2:**
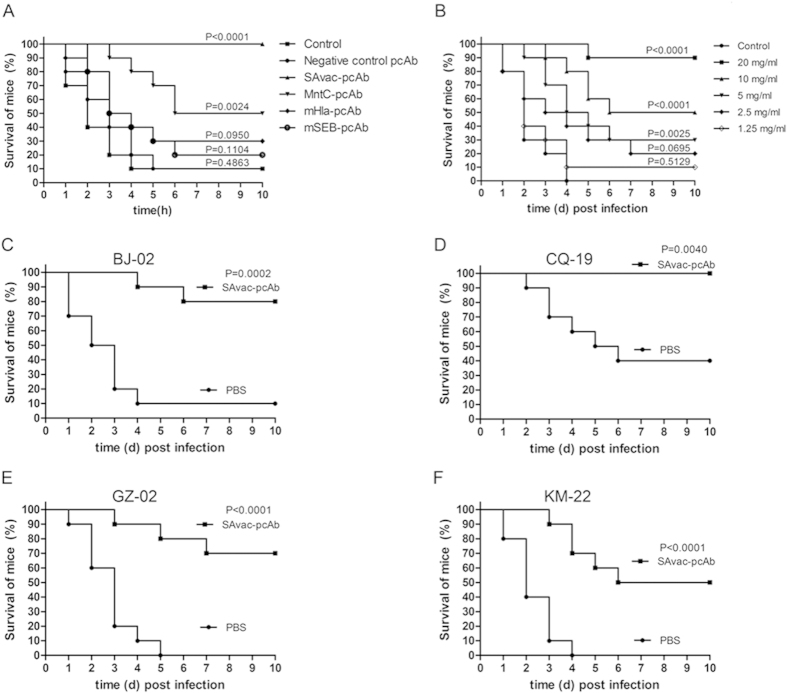
Passive immunization with pcAbs protect mice from lethal *S. aureus* challenge. Mice were infected intravenously with 100 μl of a lethal dose of *S. aureus*. 100 μl of each pcAb (20 mg/ml) was injected intravenously 2 h post infection and the survival rate for each group was monitored for 10 days after the initial infection. (**A**) Survival rates of mice (n = 10) infected with MRSA252 and passively immunized with different kind of pcAbs, as indicated. (**B**) Survival rates of mice (n = 10) infected with MRSA252 and passively immunized with different concentration of SAvac-pcAb, as indicated. (**C**–**F**) Survival rates of mice (n = 10) infected with different clinical strains of *S. aureus* and passively immunized with SAvac-pcAb. The differences between vaccinated and PBS control mice were presented as p-value.

We next examined the protective efficacy of passive immunization with pcAbs generated above in a murine sepsis model. As shown in Fig. 2A, mice (n = 10) immunized with mHla-pcAb, mSEB-pcAb, MntC-pcAb, and SAvac-pcAb all displayed higher survival rates (30%, 20%, 50% and 100%, respectively) as compared to the PBS control group (10% survival). The mice that were passively immunized with SAvac-pcAb exhibited the highest survival rate, which was significantly higher than any other group (P < 0.05), with an ED_50_ of 7.37 mg/ml as calculated by the survival rates from mice immunized with different concentrations of SAvac-pcAb (Fig. 2B). For the other three experimental groups immunized with antibodies generated using only a single antigen, mice immunized with MntC-pcAb exhibited the highest survival rate when compared to subjects immunized with either mHla-pcAb or mSEB-pcAb. In addition, passive immunization prolonged the survival time of dead mice. In the PBS control group, mice did not survive past 4 days post-infection, Contrastingly, immunized mice exhibited survival times nearly 6 days post-infection. Further, higher survival rate was not observed in mice that were immunized with negative control pcAb compared with mice that were immunized with PBS, indicating that the protection efficacy observed was directly provided by antigen specific pcAbs.

As the amino acid sequences of Hla, SEB, and MntC were highly conserved among MRSA252 and different clinical strains (Supplementary Fig. 1–3). We wanted to know if pcAb generated by proteins from MRSA252 could provide protective immunity against challenge with different clinical strains of *S. aureus*. In this study, 4 clinical strains were chosen from a library that was established by our Lab based on their representation and diversity. The library contains more than 300 clinical isolates of *S. aureus* collected from different districts of China. The information of the 4 isolates was listed in supplementary table 1. As shown in Fig. 2C–F, all the 4 clinical strains exhibited different pathogenicity and virulence in mice, as indicated by the survival rates in the PBS group. SAvac-pcAb was able to protect 60% to 70% of mice from the clinical isolates challenge when compared to PBS controls. These results strongly indicated that SAvac-pcAb can provide cross-protective efficacy in mice challenged by different clinical strains of *S. aureus*.

### Passive immunization with SAvac-pcAb correlates with reduced bacterial burden and pathology

**Figure 3:**
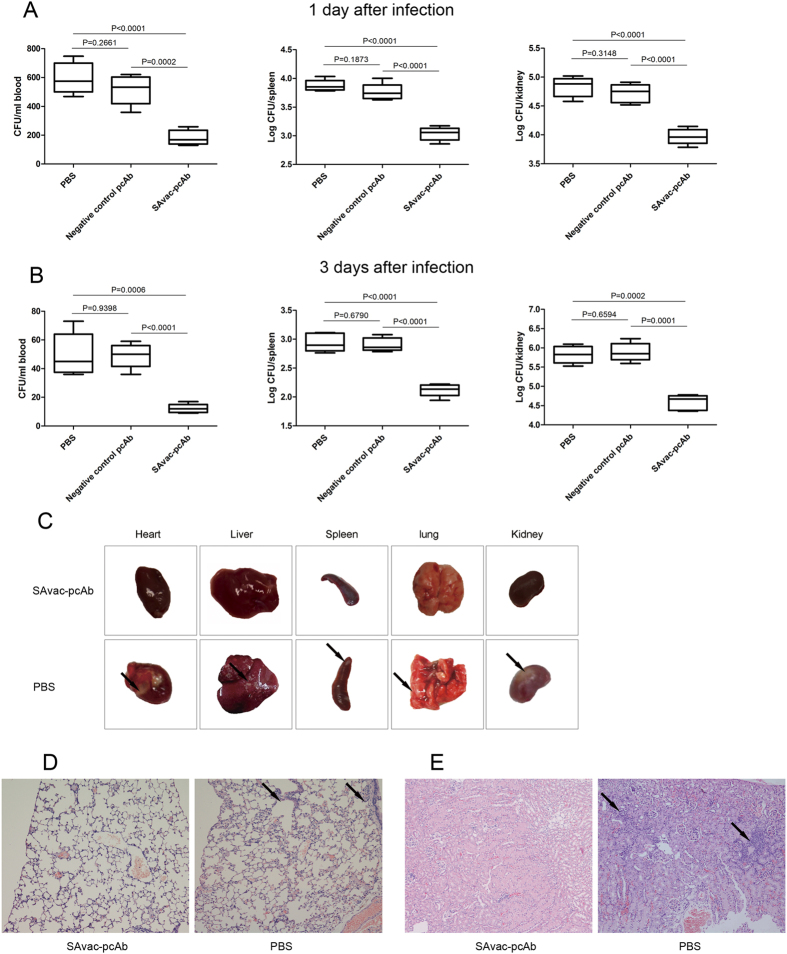
Passive immunization with SAvac-pcAb correlates with reduced bacterial burden and decreased pathology. (**A**,**B**) Bacterial loads in the blood, spleens, and kidneys of mice intravenously immunized with SAvac-pcAb, negative control pcAb and PBS were determined 1 (**A**) and 3 days (**B**) after infection with 5 × 10^8^ CFU of MRSA252. Each group includeed 5 mice. Data were presented as box plots, with the median and interquartile ranges as indicated. The differences between vaccinated and control mice were indicated as p-value. (**C**) Gross pathology of different organs from mice immunized with SAvac-pcAb and PBS with representative figures from 5 mice per group were shown. Arrows indicate typical staphylococcal abscesses in the organs. (**D**,**E**) Hematoxylin-eosin-stained kidneys (**D**) and lung (**E**) from mice immunized with SAvac-pcAb and PBS. 3 days after infection and immunization, kidneys and lungs were harvested and stained. Representative histopathological sections from 5 mice per group were shown (magnification = 100 X). Arrows indicated inflammatory cell infiltration.

The blood, spleens, and kidneys from immunized and control mice were harvested and bacterial burden at 1 and 3 days post MRSA252 infection was then evaluated. As shown in Fig. 3A,B, mice that were passively immunized with SAvac-pcAb showed lower bacterial loads of *S. aureus* in the selected organs than those from PBS controls. This decrease in load was evident at both 1 and 3 days after infection. These results suggested that SAvac-pcAb was able to partially inhibit *S. aureus* growth and colonization *in vivo*. Meanwhile, bacterial loads in mice that were passively immunized with negative control pcAb were similar as that immunized with PBS, indicating that antigen specific pcAbs directly correlated with reduced bacterial burden rather than by nonspecific stimulation of immune responses by foreign antibody.

Passive immunization with SAvac-pcAb also reduced the pathology of *S. aureus* infection. Representative gross pathology of different organs from immunized or PBS control mice were collected and imaged. No significant pathological changes were observed in SAvac-pcAb immunized mice. However, typical staphylococcal abscesses were observed in mice immunized with PBS (Fig. 3C). Consistently, histological analysis showed that mice immunized with SAvac-pcAb exhibited reduced inflammatory cell infiltration, bleeding, and tissue damage when compared to PBS group (Fig. 3D,E). These results further confirmed a protective efficacy of SAvac-pcAb *in vivo*.

### Neutrophils, but not macrophages, are essential for SAvac-pcAb induced protection and are able to kill *S. aureus in vitro*

**Figure 4:**
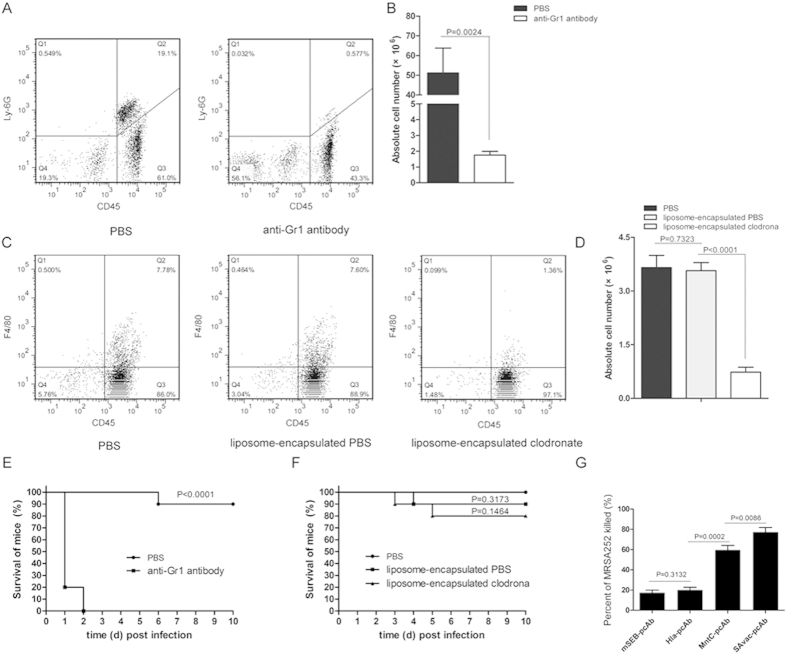
Neutrophils are involved in SAvac-pcAb induced protection against lethal *S. aureus* challenge. (**A**,**B**) Neutrophils in BALB/c mice (n = 5) were depleted using anti-Gr1 antibody as previously described. Flow cytometry assay indicated that the percentage (**A**) and absolute number (**B**) of neutrophils in depleted mice were significantly reduced when compared to PBS-treated group. (**C**,**D**) Macrophages in BALB/c mice were depleted with liposome-encapsulated clodronate, flow cytometry assay indicated that percentage (**C**) and absolute numbers (**D**) of macrophages in the liposome-encapsulated clodronate group were significantly reduced when compared to liposome-encapsulated PBS, whereas the latter exhibited no difference when compared to PBS controls. (**E**,**F**) Survival rates of neutrophil (**E**) and macrophage (**F**) depleted or control mice (n = 10) were infected with MRSA252 and passively immunized with SAvac-pcAb. The differences between vaccinated and control mice were presented as p-value. (**G**) Opsonophagocytic killing assay, MRSA252 was incubated in the presence of isolated 4 × 10^5^ HL-60 cells and pcAb in the presence of infant rabbit complement for 2 h at 37 °C, and plated on agar medium and incubated for 24 h before measuring bacterial survival as determined by CFU. The percentage of killing was calculated to determine killing activity. Data shown were means ± standard deviation (SD) derived from three independent experiments. The differences between each group were presented as p-value.

Neutrophils and macrophages are the two major types of phagocytes found in peripheral blood that function in antibody-mediated opsonophagocytosis^16^. Given their importance, we sought to determine which cell type was essential for the SAvac-pcAb mediated protection observed during *S. aureus* challenge. Neutrophils and macrophages in BALB/c mice were depleted using an anti-Gr1 antibody and liposome-encapsulated clodronate, respectively. As shown in Fig. 4A–D, flow cytometry assay indicated that the absolute number and percentage of neutrophils and macrophages in depleted mice were significantly reduced as compared to control mice, with a deletion rate of 96.6% and 80.0% for neutrophils and macrophages, respectively.

After MRSA252 challenge and subsequent passive immunization with SAvac-pcAb, all neutrophil-depleted mice (n = 10) died within two days. This was significantly different from those in the PBS control group (10%, P < 0.0001) (Fig. 4C), contrastingly, macrophage-depleted mice showed no difference in survival rates when compared to either PBS or liposome-encapsulated PBS group (Fig. 4D). These results indicated that neutrophils were critical in SAvac-pcAb mediated protection against *S. aureus* infection.

We then carried out an *in vitro* opsonophagocytic killing assay to confirm the protective role of neutrophils. In the presence of differentiated HL-60 cells and rabbit serum complement, all 4 pcAbs exhibited increased opsonophagocytic activity for MRSA252. SAvac-pcAb was able to effectively kill more than 70% of the bacteria, which was significantly higher than the other 3 pcAbs. In addition, MntC-pcAb exhibited a higher opsonophagocytic activity as compared to either mHla-pcAb or mSEB-pcAb. No differences were observed between the latter two (Fig. 4E). These results indicated (i) SAvac-pcAb was able to kill MRSA252 cells efficiently *in vitro* and (ii) MntC-specific IgG might be the major antibody involved in neutrophil-dependent, opsonophagocytic killing.

### pcAbs are able to directly recognize MRSA252 *in vitro*

**Figure 5:**
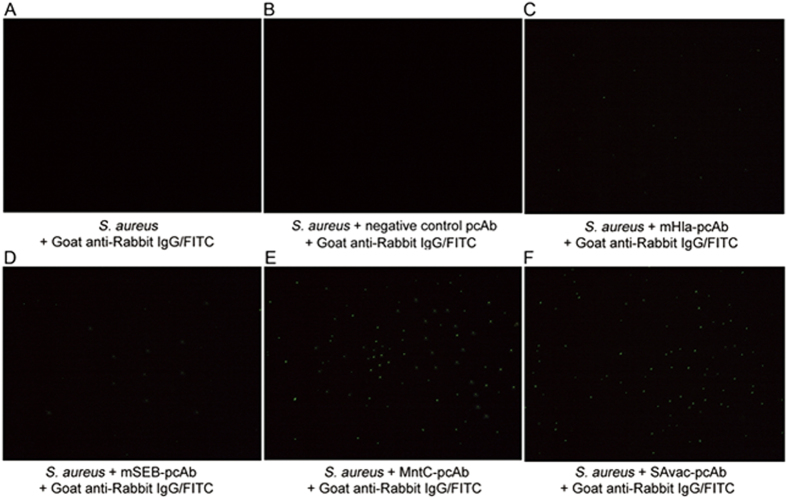
pcAbs are able to directly recognize MRSA252 *in vitro*. Indirect immunofluorescence was conducted to confirm the binding of pcAb to MRSA252 *in vitro*. No immunofluorescence was detected in the absence of pcAb (**A**,**B**). Positive, indirect immunofluorescence signals indicated binding of different pcAb, as indicated, with MRSA252 (**C–F**). This study was performed twice, with similar results.

Since all the antigens used to generate pcAb in this study were surface exposed (MntC) or secreted (SEB and Hla) proteins, we wondered if specific antibodies generated by these antigens could directly recognize *S. aureus in vitro*. In this study, indirect immunofluorescence assay was carried out to validate the interaction between pcAb and MRSA252. The results clearly indicated that no immunofluorescence was detected in the absence of antigen specific pcAbs (Fig. 5A,B), whereas positive indirect immunofluorescence signals indicated the binding of four kinds of pcAb with MRSA252 (Fig. 5C–F). An increase in fluorescence signal intensity was observed in MntC-pcAb when compared to the mHla-pcAb and mSEB-pcAb group, this could be explained by the fact that MntC is a surface exposed protein while the other two are secreted toxins, and this result was consistent with our previous result that MntC-pcAb exhibited higher opsonophagocytic activity when compared to mHla-pcAb or mSEB-pcAb. Meanwhile, the fluorescence signal intensity was similar in SAvac-pcAb and MntC-pcAb group (Fig. 5E,F). Collectively, these results indicated that all pcAbs generated in this study were able to bind MRSA252 *in vitro* and MntC-pcAb was the most important antibody involved, and the binding of pcAbs with MRSA252 may essential in clearing and controlling the infection.

### pcAbs inhibit the biological function of Hla and SEB *in vitro*

**Figure 6:**
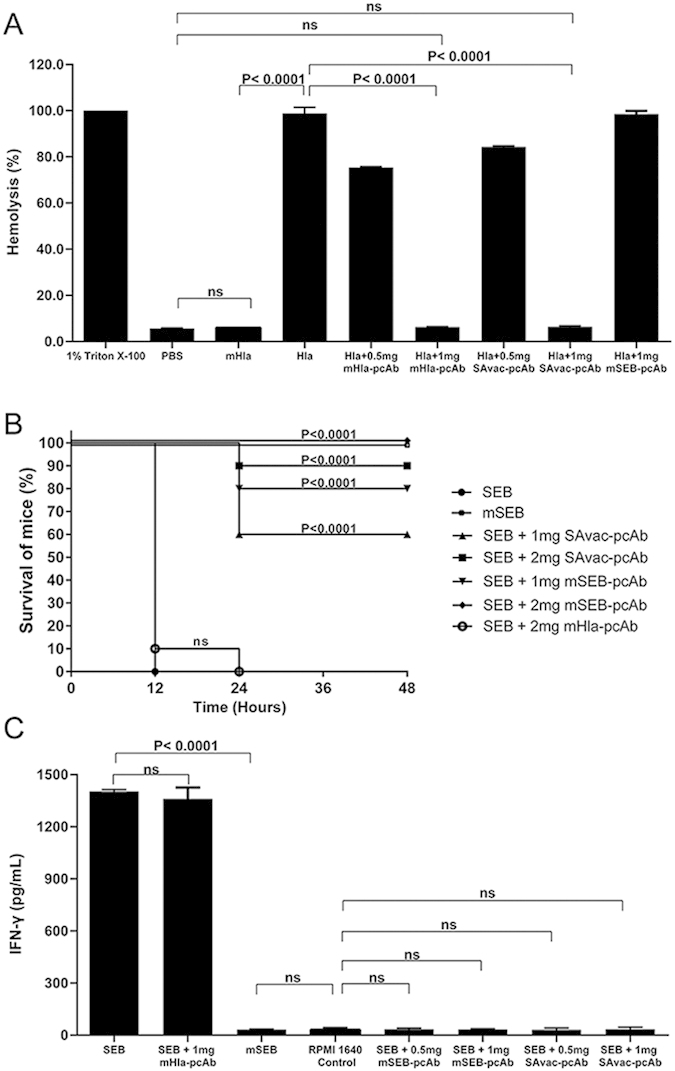
pcAb inhibits the biological function of Hla and SEB *in vitro*. (**A**) Hemolytic activity assay, 1% rabbit erythrocytes diluted with PBS were incubated at 37 °C for 30 min with mHla, Hla, or Hla pre-incubated with mHla-pcAb or SAvac-pcAb, as indicated. The supernatant was then isolated by centrifugation and the hemolytic activity was determined by the release of hemoglobin, measured spectrophotometrically at 540 nm and presented as % hemolysis of the positive control (Triton X-100). Experiments were conducted in triplicate and data were presented as mean values ± standard deviation (SD). (**B**) Intestinal toxin activity assay, mice were injected i.p. with D-Galactosamine (200 mg/mL) followed by an intramuscular injection with the indicated concentrations of SEB, mSEB, PBS, or SEB incubated with mSEB-pcAb and SAvac-pcAb for 30 min prior to the start of the study. Survival was monitored for 40 h after the challenge and mHla-pcAb was used as a control, the differences between each group were presented as p-value. (**C**) IFN-γ production assay. Pig splenic leukocytes were isolated and cultured in the presence of the indicated concentrations of wild type SEB or mSEB. After 40 h of culture, supernatants were taken and ELISA was used to determine IFN-γ secretion. mHla-pcAb was used as control and data were presented as mean values ± standard deviation (SD). Experiments were conducted in triplicate.

Hla functions as a hemolysin and is an important virulence factor in *S. aureus*^17^. We therefore examined the inhibitory efficacy of mHla-pcAb and SAvac-pcAb on hemolysis of rabbit erythrocyte by Hla. As shown in Fig. 6A, 10 μg of Hla exhibited similar hemolytic activity as compared to the positive control group (1% Triton X-100). In contrast, incubation of mHla-pcAb or SAvac-pcAb with Hla resulted in a dose-dependent inhibition of hemolytic activity. Further, no significant differences in hemolysis activity were observed when Hla was incubated with 1 mg of mHla-pcAb or SAvac-pcAb as compared to the negative control group, indicating that 1 mg of these antibodies was sufficient to completely inhibit the hemolytic activity of 10 μg Hla, which might be essential in SAvac-pcAb mediated protection during *S. aureus* infection.

Intestinal toxin activity is the most important biological function of SEB and it is directly involved in the pathogenicity of *S. aureus*^18^. We evaluated the impact of mSEB-pcAb and SAvac-pcAb on intestinal toxin activity of wild type SEB. After sensitizing with D-Galactosamine, all mice that were challenged with wild type SEB (25 μg) died within 24 h. In contrast, no death was observed in those subjects challenged with 0.2 mg of mSEB. In addition, incubation of SEB with either mSEB-pcAb or SAvac-pcAb significantly increased the survival rate in a dose dependent manner (Fig. 6B). These results confirm that SAvac-pcAb was able to inhibit the intestinal toxin activity of SEB efficiently.

SEB is also a super-antigen that is capable of robustly increasing IFN-γ production by mononuclear leukocytes^19^. As such, we determined the impact of mSEB-pcAb and SAvac-pcAb on SEB-mediated IFN-γ production by swine mononuclear leukocytes. As shown in Fig. 6C, compared to negative control, 10 μg of SEB significantly increased the secretion of IFN-γ (P < 0.0001), while the same amount of mSEB showed no difference. Furthermore, incubation of SEB with 0.5 or 1 mg of mSEB-pcAb or SAvac-pcAb resulted in a dramatic reduction of IFN-γ production. This was significantly different compared to SEB injection alone (P < 0.0001). These observations confirmed the inhibitory role of anti-mSEB antibody on the super-antigen activity of SEB.

## Discussion

Passive immunization has long been used as an effective treatment option for either bacterial or viral infectious diseases^20^,^21^,^22^,^23^. Typically, three categories of antibodies were used for passive immunization, including (i) monoclonal antibody or its genetically modified derivatives^24^,^25^, (ii) Homologous or heterologous serum containing pcAb^26^, and (iii) IgG purified from pcAb (pcAb-IgG)^20^,^27^. In this study, pcAb-IgG was used for the treatment of *S. aureus* infection, as most of extra serum components that may inducing side effects were removed before immunization, it was safer than the other two categories of antibodies and exhibit higher specificity.

The efficacy of pcAb-IgG relies in large part on antigens used for the generation of pcAb. The protective efficacy of three antigens chosen in this study has been studied elsewhere^8^,^10^,^11^,^15^. Hla is a major virulence factor in *S. aureus* and is lethal to rodents and rabbits^28^, vaccination against Hla protects mice from lethal *S. aureus* pneumonia^29^. SEB is a *S. aureus* exotoxins and is directly associated with staphylococcal Toxic Shock Syndrome (TSS)^30^, SEB-specific monoclonal antibodies is able to partially protect against TSS^18^ and *S. aureus* infection^31^. MntC, a lipoprotein that is involved in the uptake of manganese ions^32^, is a highly conserved cell surface protein that elicits protective immunity against *S. aureus*^33^. Thus, all of the three chosen proteins are either key virulence factors of *S. aureus* or directly involved in bacterial metabolism, this range of antigens allows us the possibility of greatest infection control by blocking the biological function of these targets.

Antibodies function in various ways, as some can directly neutralize pathogens or secrete toxins, whereas others can evoke potent antibody-dependent cell-mediated cytotoxicity or complement dependent cytotoxicity^34^. As validated in both *in vitro* and *in vivo* studies, the efficacy of SAvac-pcAb lies in four features. (i) The antibody induced by MntC significantly promoted the ability of neutrophils in opsonophagocytic killing of *S. aureus.* (ii) SEB-specific neutralizing antibody functionally inhibited the intestinal toxin activity and super-antigen activity of the protein, thus preventing both TSS and cytokine storm, which were essential in death caused by *S. aureus* infection^35^. (iii) Hla specific antibody was able to block the hemolytic activity of Hla. (iv) SAvac-pcAb was induced by combined immunization and was able to simultaneously target multiple pathogenic pathways of *S. aureus*. As indicated in our results, an antibody induced by just one antigen provideed only partial protection (<50%), whereas combined immunization with SAvac-pcAb significantly enhanced overall protective efficacy (>90%).

Two categories of peripheral cells, neutrophils and macrophages, are the major cell types that function in antibody-mediated opsonophagocytosis. Li *et al*^36^ reported that Sao-induced antibodies significantly promoted the ability of porcine neutrophils in opsonophagocytic killing of *S. suis*, whereas a separate research group proposed that macrophages were essential for the killing of group B streptococci by opsonic antibody^36^. In our study, both *in vitro* and *in vivo* results emphasized the importance of neutrophils in the SAvac-pcAb mediated killing of *S. aureus*. However, the mechanism by which cell type is involved in antibody-mediated opsonophagocytic killing remains unclear, but likely includes many factors such as the subtype of the antibody.

Taken together, the results obtained in the present study demonstrated that therapeutic use of SAvac-pcAb resulting in reduced bacterial loads and improved protection in a sepsis model of lethal *S. aureus* infection. Antibody-mediated opsonophagocytosis by neutrophils, in combination with neutralizing activity against Hla and SEB, were essential for the high protective efficacy we observed. Since *S. aureus* has posed therapeutic challenges world-wide, and some strains become resistant to nearly all front-line antibiotics, our results provided the basis for an alternative therapeutic strategy for the treatment of *S. aureus* infection by pcAb-mediated immunotherapy.

## Methods

### Ethics statement

All animal care and use protocols in this study were performed in accordance with the Regulations for the Administration of Affairs Concerning Experimental Animals approved by the State Council of People's Republic of China. All animal experiments in this study were approved by the Animal Ethical and Experimental Committee of the Third Military Medical University (Chongqing, Permit No. 2011-04) in accordance with their rules and regulations. All surgery was performed under sodium pentobarbital anesthesia, and all efforts were made to minimize suffering.

### Plasmids, proteins, bacterial strains, and culture method

Plasmids containing the sequence encoding wild type (Hla, SEB and MntC) and mutated (mHla, mSEB) proteins were constructed and maintained in our lab. Proteins were expressed and purified as described previously^10^,^11^,^37^. The *S. aureus* standard strain MRSA252 was purchased from ATCC (Manassas, VA, USA). Clinical strains of 4 *S. aureus* isolates were collected from 4 hospitals in different districts of China (Supplementary Table 1). Bacterial strains were cultured in tryptic soy broth, washed and diluted with sterile PBS to an appropriate cell concentration determined spectrophotometrically at 600 nm (OD600).

### Production, purification, and characterization of pcAb

pcAbs were generated in New Zealand white rabbits based on a previously published method^38^. The IgG antibodies in the serum from antigen immunized or unimmunized rabbits were purified by affinity chromatography with a protein A column (GE, USA), followed by desalting with PBS, the concentration of each pcAb was determined by the BCA method^39^ and adjusted to a final concentration of 20 mg/ml. The titer of the antigen-specific IgG antibodies in each pcAb was determined by ELISA using Maxisorp microtiter plates that had been previously coated with each recombinant protein (400 ng/well) or SA-WCL (1 μg/well). The antibody titer was defined as the highest dilution with an absorbance value (OD450) of more than twice the blank control.

### Immunization and bacterial challenge

Six- to eight-week-old female BALB/c mice were randomized into each group (n = 10), and infected intravenously with 100 μl of either a lethal dose of MRSA252 (1 × 10^9^ CFU per mouse) or one of the 4 clinical strains (3 × 10^9^ CFU per mouse). 2 h after infection, mice were passively immunized intravenously with 100 μl of each pcAb (2 mg) or the same volume of PBS for control subjects. The survival rates for all mice were monitored for 10 days after infection. To determine the ED_50_ of SAvac-pcAb, it was diluted to 5 different concentrations at 2-fold serial dilution and used for passive immunization. The ED_50_ was calculated by the Bliss method according to the survival rates for each group using SPSS13.0.

### Bacterial burden and histopathological analysis

Mice were infected with 5 × 10^8^ CFU of MRSA252 followed by passive immunization with 2 mg of SAvac-pcAb, 2 mg of negative control pcAb or the same volume of PBS. The blood, spleens, and kidneys were harvested and assessed for bacterial colonization at 1 and 3 days after infection. To calculate the bacterial number in each of the organs, organ homogenates were prepared in PBS and plated at 10-fold serial dilutions on tryptic soy agar. The colonies were quantified after 24 h of incubation at 37 °C. Histopathological analysis was conducted on day 14 after infection. Organs were fixed with 10% phosphate-buffered formalin and embedded in paraffin. Four-micrometer-thick sections were prepared and stained with hematoxylin and eosin (H&E) for microscopic examination.

### Depletion of neutrophils and macrophages

To deplete neutrophils, mice were injected intraperitoneally (i.p.) with either 200 μg of anti-Gr1 antibody (volume of 100 μl, clone RB6-8C5, BioXCell, USA) or a control injection of the same volume of PBS 24 hours prior to the start of the study. Injections continued every other day throughout the duration of the study. To deplete macrophages, mice were injected intravenously with 100 μl of liposome-encapsulated clodronate (FormuMax Scientific, USA) 24 hours prior to the start of the study, PBS and liposome-encapsulated PBS were used as negative control.

### Opsonophagocytic killing assay

The method used has been previously described by Burton and Nahm^40^. Briefly, HL-60 cells (ATCC, CCL-240) were differentiated into granulocyte-like cells with the addition of 100 mM N’,N dimethylformamide (Sagon, China) to the growth medium for 4 days. The assay was performed in 96-well plates, with each well containing the following: 4 × 10^5^ HL60 cells, 10^3^ CFU of MRSA252, 100 μg of each pcAb, and 1% infant rabbit serum as a complement source in a total volume of 80 μl. After incubation at 37 °C for 2 h, samples were plated on agar medium. The opsonophagocytic killing effect was defined as a reduction in CFU after overnight growth compared with the negative control group.

### Flow cytometry

In 96-well, U-bottom plates, 1 × 10^6^ erythrocyte-depleted cells in PBS and 2% FBS were added for a total volume of 100 μl. For the detection of macrophages in mouse spleens, single cell suspensions were stained with PE/Cy7 anti-mouse CD45 and APC anti-mouse F4/80. For the detection of neutrophils in mouse blood, cells were stained with PE/Cy7 anti-mouse CD45 and APC/cy7 anti-mouse Ly-6G (Biolegend, Inc., USA). Samples were then analyzed using BD FACSArray software^TM^ on a BD FACSArray flow cytometer (BD Biosciences).

### Indirect immunofluorescence

Indirect immunofluorescence was carried out based on a method established by us previously^41^.

### Hemolytic activity assay

0.9 ml of rabbit erythrocyte suspension in PBS (1%) was mixed with 100 μl of Hla, mHla, and Hla that had been incubated with different concentrations of mHla-pcAb or SAvac-pcAb 30 min prior to the start of the study. An equal volume of 1% Triton X-100 and PBS were used as positive and negative controls, respectively. After a 30 min incubation at 37 °C, the mixtures were centrifuged at 400 × g for 10 min. The hemolytic activity was determined by the release of hemoglobin, measured spectrophotometrically at 540 nm and presented as % hemolysis of the positive control (Triton X-100).

### Intestinal toxin activity assay

six- to eight-week-old female BALB/c mice were injected i.p. with 100 μl of D-Galactosamine (200 mg/ml) followed by an intramuscular injection of 100 μl of SEB (0.25 mg/ml), mSEB (2 mg/ml), or SEB (0.25 mg/ml) that had been incubated with 1 mg or 2 mg of mSEB-pcAb or SAvac-pcAb 30 min prior to the start of the study. mHla-pcAb was used as negative control. Survival was monitored for 40 h after injection.

### IFN-γ production assay

Mononuclear leukocytes isolated from the spleen tissue of a healthy pig were counted and plated at 5 × 10^5^ per well (100 μl) before incubation with RPMI 1640 medium (Gibco, Beijing, China) supplemented with 10% fetal calf serum (Gibco, USA), then 100 μl of SEB, mSEB, or SEB (0.1 mg/ml) that had been incubated 30 min prior to the start of the study with 0.5 or 1 mg of mSEB-pcAb or SAvac-pcAb were added to each well. Culture supernatants were harvested 40 h later, and porcine IFN-γ production was quantified using by ELISA. mHla-pcAb was used as the negative control.

### Statistical analyses

Data were presented as mean ± standard deviation (SD). Means were compared using the two-tailed Student’s t-test. Mantel-Cox Log-rank analysis was utilized to determine differences in survival times. P < 0.05 was considered statistically significant.

## Additional Information

**How to cite this article**: Zhang, J. *et al.* Protective Efficacy and Mechanism of Passive Immunization with Polyclonal Antibodies in a Sepsis Model of *Staphylococcus aureus* Infection. *Sci. Rep.* **5**, 15553; doi: 10.1038/srep15553 (2015).

## Supplementary Material

Supplementary Information
