# Supplementary material for: Protective Efficacy and Mechanism of Passive Immunization with Polyclonal Antibodies in a Sepsis Model of Staphylococcus aureus Infection
Source: Sci Rep. 2015 Oct 22;5:15553. doi: 10.1038/srep15553 (PMC4614693; doi:10.1038/srep15553)
Supplement: Supplementary Information [file srep15553-s1.pdf]

Protective Efficacy and Mechanism of Passive Immunization with Polyclonal Antibodies in a Sepsis Model of *Staphylococcus aureus* Infection

Jinyong Zhang[1,+], Feng Yang[1,2,+], Xiaoli Zhang[3], Haiming Jing[1], Chunyan Ren[1], Changzhi Cai[1], Yandong Dong[1], Yudong Zhang[1], Quanming Zou[1,*], Hao Zeng[1,*]

[1]National Engineering Research Center of Immunological Products, Department of Microbiology and Biochemical Pharmacy, College of Pharmacy, Third Military Medical University, Chongqing, 400038, PR China.

[2]College of Bioengineering, Chongqing University, Chongqing, 400044, PR China

[3]Department of Clinical Hematology, Southwest Hospital, Third Military Medical University, Chongqing, 400038, PR China.

[+] These authors contribute equally to this work.

[*] Corresponding author: Quanming Zou, E-mail:qmzou2007@163.com.

Hao Zeng, E-mail:zeng1109@163.com.

Supplementary Fig. 1 Sequence alignment of Hla from MRSA252 with those from 4 clinical strains of *S. aureus*.

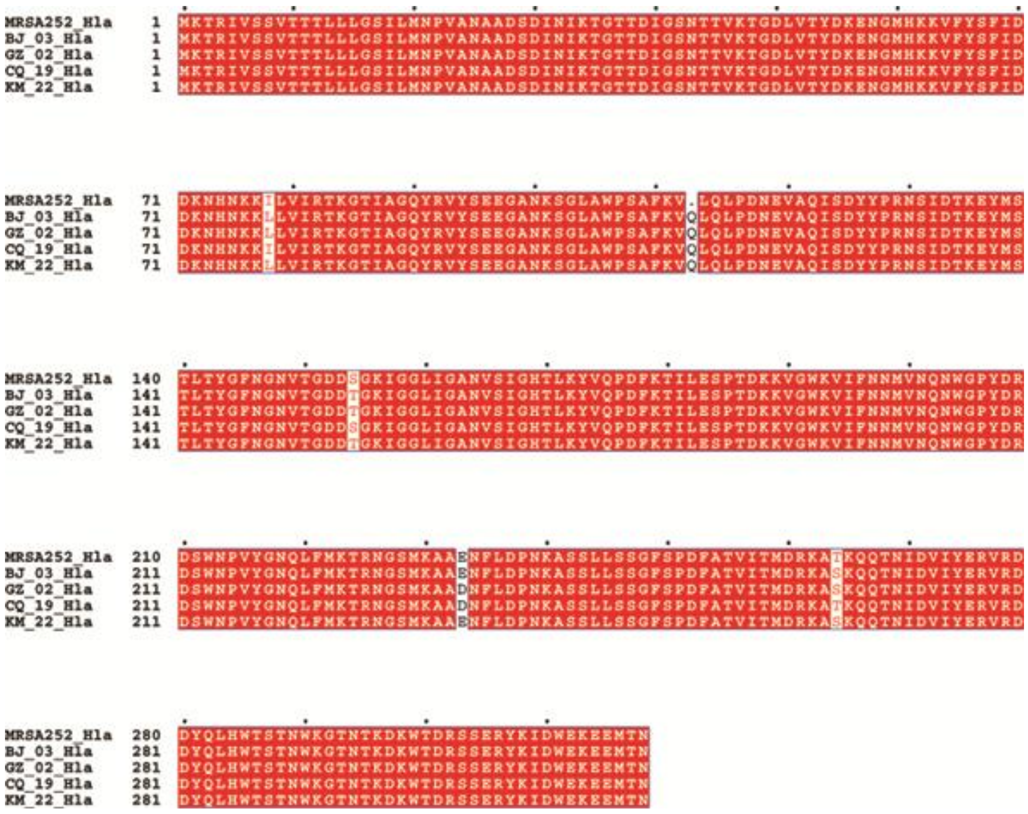

Supplementary Fig. 2 Sequence alignment of MntC from MRSA252 with those from 4 clinical strains of *S. aureus.*

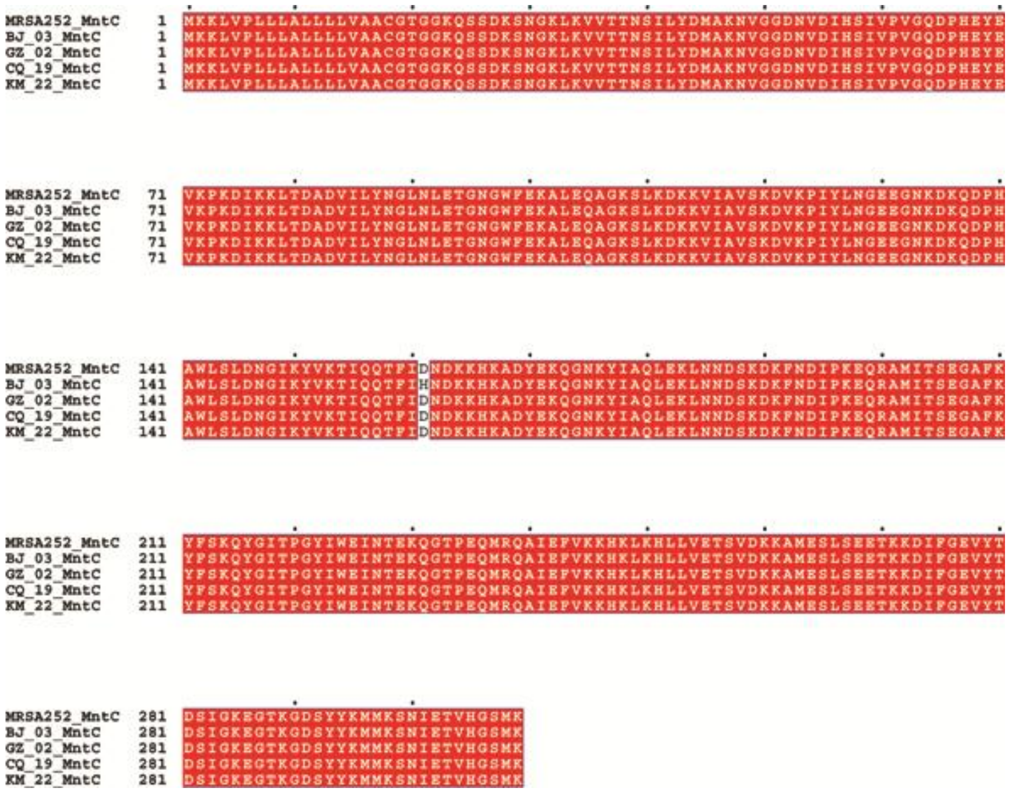

Supplementary Fig. 3 Sequence alignment of SEB from MRSA252 with those from 4 clinical strains of *S. aureus*.

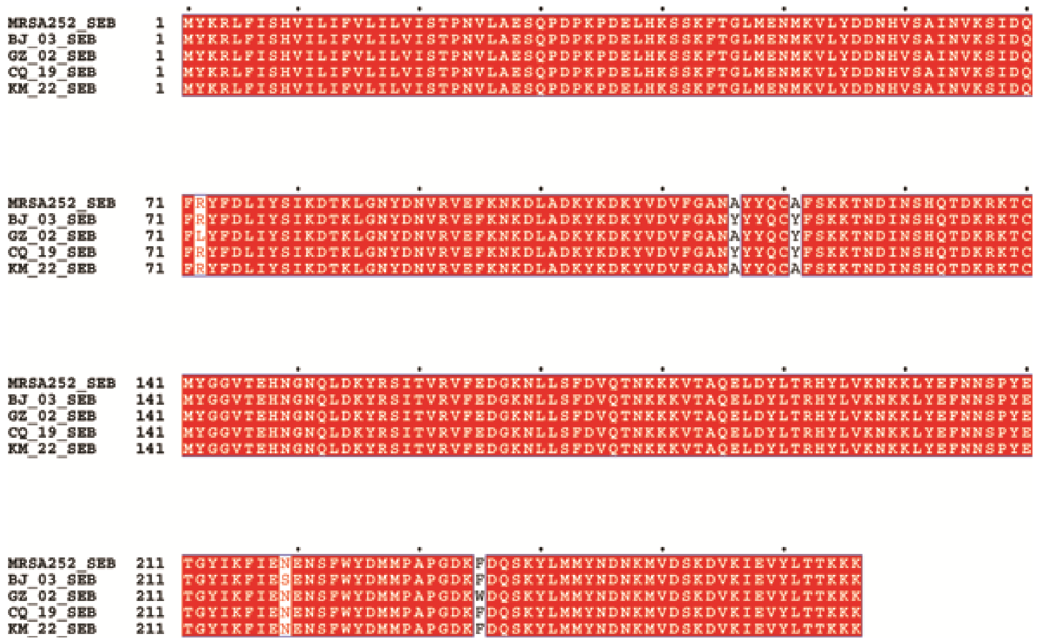

Supplementary Table 1. The information of *S. aureus* clinical strains used in this study.

Strain name	Source of strain	Department	Patient's name	Gender	Age	Source of sample	Nuc gene[#]	mecA gene[*]	Genotyping[&]
BJ-02	Beijing 263 hospital	Respiratory	Zhang××	Female	69	Sputum	+	+	ST239-t437-SCCmecIII
CQ-19	Chongqing southwest hospital	ICU	Liu×	Female	22	Blood	+	+	ST398-t030-SCCmecIII
GZ-02	Guangzhou nanfang hospital	Traumatic fracture	Zhang×	male	21	Puncture fluid	+	+	ST5-t037-SCCmecIV
KM-22	Kunming medical college	Burns surgery	Lu××	male	4	Secretions	+	-	ST121-t030

[#] nuc gene, which encode the thermonuclease, has been widely used as a specific marker to detect *S. aureus* by PCR-based methods.

[*] The product of mecA gene is penicillin-binding protein, which resulting in multiple drug resistance.

[&] Three Genotyping methods were used in this study, including the multiple locus sequence typing (ST), Staphylococal Protein A genetic polymorphism analysis (t) and classification of Staphylococcal cassette chromosome mec (SCCmec).
